# Limited roles of the miR‐17‐92 cluster in the regulation of T‐cell apoptosis

**DOI:** 10.1111/febs.70387

**Published:** 2026-01-06

**Authors:** Katharina Hoppe, Johannes Woelk, Julia Benz, Sarah Spoeck, William J. Olson, Andreas Villunger, Natascha Hermann‐Kleiter, Verena Labi

**Affiliations:** ^1^ Institute for Developmental Immunology, Biocenter Medical University of Innsbruck Austria; ^2^ Department for Genetics and Pharmacology Institute of Cell Genetics, Medical University of Innsbruck Austria; ^3^ Institute for Biomedical Aging Research University of Innsbruck Austria; ^4^ The Research Center for Molecular Medicine (CeMM) of the Austrian Academy of Sciences Vienna Austria

**Keywords:** apoptosis, BIM, miR‐17‐92, T cells

## Abstract

The pro‐apoptotic BCL‐2‐interacting mediator of cell death [BIM; also known as Bcl‐2‐like protein 11 (BCL2L11)] is a crucial regulator of programmed cell death in immune cells, with roles in T‐cell development, homeostasis, and immune response modulation. However, the precise molecular mechanisms that regulate BIM expression in these processes are not completely understood. One possible regulatory mechanism involves microRNAs, small noncoding RNAs that silence target messenger RNAs (mRNAs). The miR‐17‐92 cluster, which has been implicated in immune regulation, has nine predicted binding sites in the 3′ untranslated region of the *Bcl2l11* mRNA (thereafter referred to as *Bim* mRNA). To explore whether direct miR‐17‐92‐mediated regulation of BIM controls apoptosis in T cells, a genetically modified mouse model with disrupted miR‐17‐92:Bim interactions specifically in T cells has been used. The results revealed that loss of miR‐17‐92:Bim binding, although leading to a modest increase of BIM protein in double‐positive (DP) thymocytes and naïve CD8^+^ T cells, does not measurably affect early T‐cell development or peripheral T‐cell numbers. However, the absence of this interaction led to a moderate reduction in Th_17_ CD4^+^ T cells at a steady state. Collectively, these findings suggest that miR‐17‐92‐mediated regulation of BIM does not play major roles in T‐cell apoptosis and homeostasis, highlighting the existence of alternative regulatory mechanisms controlling BIM pro‐apoptotic activity.

## Graphical abstract

The pro‐apoptotic protein BIM shapes T‐cell survival and function, but how it is regulated remains unclear. In a mouse model lacking direct miR‐17‐92:Bim interactions, we observed a moderate reduction in Th_17_ CD4^+^ T cells. BIM levels were modestly increased in double‐positive thymocytes and naive CD8^+^ T cells, but this did not affect overall T‐cell development or homeostasis. Our findings point to alternative pathways controlling BIM‐driven apoptosis in T cells. Created with BioRender.com.

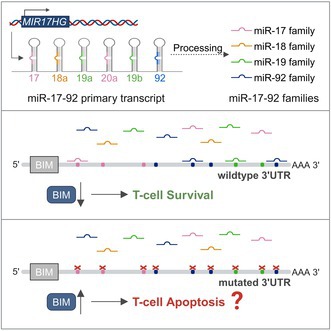

**Abbreviations**
3′ UTR: 3′ untranslated region
BIM: BCL‐2 interacting mediator of cell death
CM: central memory
Dex: dexamethasone
DN: double negative
DP: double positive
EM: effector memory
miRNA: microRNA
MPEC: memory precursor effector cell
mRNA: messenger RNA
N: naive
nt: nucleotide
SLEC: short‐lived effector cell
SP: single positive
TCR: T‐cell receptor
Tfh: T follicular helper cell
Th: T helper cell
Treg: regulatory T cell

## Introduction

T cells are a critical component of immunity to pathogens or tumors. Programmed cell death (apoptosis) is crucial for proper T‐cell development in the thymus and homeostasis in the periphery, while preventing aberrant T‐cell responses, autoimmunity or neoplasia [1]. Notably, the pro‐apoptotic BCL‐2 family member BIM (BCL‐2 interacting mediator of cell death) serves major and nonredundant roles among BH3‐only proteins in shaping a functional T‐cell compartment. During T‐cell development, BIM‐mediated apoptosis efficiently eliminates autoreactive CD4^−^CD8^−^ double‐negative (DN) thymocytes during negative selection [2, 3], restricts the agonist‐selected thymocyte pool [4, 5], and mediates apoptosis upon nonresponsiveness to IL‐7 signaling [6]. In naïve recirculating T cells, BIM determines the lifespan of both CD4^+^ and CD8^+^ T cells [2, 7, 8]. Finally, BIM‐mediated apoptosis is key for a timely contraction of the expanded pool of activated CD4^+^ and CD8^+^ T cells after the peak of the immune response [7, 9, 10, 11, 12, 13, 14, 15]. Intriguingly, CD8^+^ effector cells with high BIM and high BCL2 levels have a greater memory potential [11, 16], suggesting that apoptosis may, beyond eliminating superfluous cells, actively shape the memory cell pool. Further, BIM mediates the apoptosis of effector and memory CD4^+^ T cells, most notably IFNγ‐producing T helper (Th) 1 effector cells [15, 17, 18], and limits the pool of thymic and peripheral regulatory T cells (T_reg_) [19, 20]. To prevent aberrant apoptosis while ensuring tissue homeostasis, BIM expression requires precise control, which can be exerted at the transcriptional, post‐transcriptional, and post‐translational level. However, although it has been established that signaling from cytokine receptors and the (pre) T‐cell receptor (TCR) impact BIM expression in T cells, surprisingly little is known on direct control mechanisms of BIM levels.

microRNAs (miRNAs) are post‐transcriptional regulators of gene expression. ~22 nt in length, they function by binding to mRNAs, mainly in their 3′ untranslated region (3′ UTR), in a sequence‐specific manner [21]. Genetic studies in mice have revealed that the miRNA families miR‐17, miR‐18, miR‐19, and miR‐92, encoded by the polycistronic miR‐17‐92 cluster and its 2 paralogs, miR‐106a‐363 and miR‐106b‐25, impact profoundly on various aspects of T‐cell biology [22, 23, 24, 25, 26, 27, 28, 29, 30, 31, 32]. In DN thymocytes, miR‐17‐92 miRNAs promote proliferation and inhibit apoptosis downstream of IL‐7 and dampen pre‐TCR signaling‐mediated apoptosis during negative selection [30, 32]. Whereas little is known about the impact of miR‐17‐92 on the lifespan of mature naïve T cells, a series of reports describes roles upon T‐cell activation. In CD8^+^ T cells, miR‐17‐92 promotes the proliferation and differentiation of short‐lived effector cells, while downregulation of miR‐17‐92 is necessary for the optimal development of memory precursor effector cells [33, 34]. In CD4^+^ T cells, miR‐17‐92 facilitates differentiation to Th_17_ [35] and IFNγ‐producing Th_1_ cells [36] at the expense of T_reg_ differentiation [35, 36]. Major roles have been assigned to miR‐17‐92 in T follicular helper (T_fh_) cell differentiation [28, 29]. Of note, mice overexpressing miR‐17‐92 in lymphocytes develop lymphoproliferation and autoimmunity due to aberrant cell survival [24].

Given the opposing roles of BIM and miR‐17‐92 in cell survival, we investigated whether miR‐17‐92 may exert, at least partially, its functions in T cells via repression of BIM. Of note, loss of BIM prevents the reduction in T and other immune cells that is caused by acute deletion of miR‐17‐92 in mice [37]. However, it is not clear in which physiological settings miR‐17‐92:Bim interactions are indeed functional. Together with PTEN, BIM has emerged as a major joint miR‐17‐92 target gene, whose mRNA harbors nine putative binding sites for the miR‐17, miR‐19, and miR‐92 families, making it a strong candidate target gene. However, although direct miRNA:mRNA binding may result in mRNA decay or translation inhibition and consequently reduced protein expression, miRNA binding to mRNAs often does not lead to a change in protein levels. Therefore, we used a previously published mouse model, which would allow us to specifically abolish miR‐17‐92:Bim binding in T cells [38]. PAR‐CLIP showed that binding sites used in wild‐type cells were no longer covered by miR‐17‐92 in cells from mutant mice. Of note, abolishing this interaction in the germline caused neonatal lethality due to a lung developmental defect [38].

Ablating miR‐17‐92:Bim binding from early T‐cell development in the thymus, we found that thymocytes do not rely on this interaction for their survival at various developmental checkpoints. Although mature naïve T‐cell numbers were not altered in the periphery of these mice, CD8^+^ naïve T cells had slightly elevated BIM levels, indicating functional interactions between miR‐17‐92 and its target and suggesting potential roles in the lifespan of these cells. *In vivo*, we did not find alterations in the fractions of CD8^+^ short‐lived effector cells or memory precursor effector cells at steady state. However, *in vitro* cultures showed that early upon stimulation, miR‐17‐92:Bim binding selected for IFNγ^hi^ cells within this population, suggesting enhanced functional properties. Finally, the absence of this interaction leads to a significant reduction in Th_17_ CD4^+^ T cells *in vivo* at steady state, indicating protection of differentiating Th_17_ cells by miR‐17‐92 from BIM‐mediated apoptosis. Overall, however, our findings suggest that miR‐17‐92‐mediated regulation of BIM is not detrimental to T‐cell apoptosis and homeostasis at steady state.

## Results

### Both miR‐17‐92 and *Bim* are expressed throughout the T‐cell compartment

**Fig. 1:**
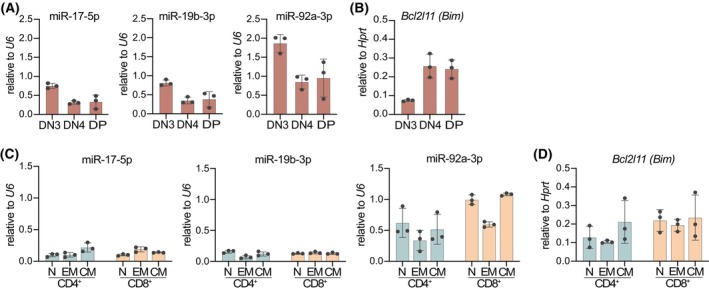
Both miR‐17‐92 and *Bim* are expressed throughout the T‐cell compartment. (A) qRT‐PCR analyses of miR‐17‐5p, miR‐19b‐3p, and miR‐92a‐3p expression in FACS‐sorted double‐negative (DN)3, DN4, and double‐positive (DP) thymocytes from wild‐type mice (*n* = 3; data are presented as mean ± SD). (B) qRT‐PCR analyses of *Bcl2I11 (Bim)* in FACS‐sorted double‐negative (DN)3, DN4, and double‐positive (DP) thymocytes from wild‐type mice (*n* = 3; data are presented as mean ± SD). (C) qRT‐PCR analyses of miR‐17‐5p, miR‐19b‐3p, and miR‐92a‐3p expression in FACS‐sorted naïve (N), effector memory (EM), and central memory (CM) CD4^+^ and CD8^+^ T cells from spleen and lymph node pools of wild‐type mice (*n* = 3; data are presented as mean ± SD). (D) qRT‐PCR analyses of *Bcl2I11 (Bim)* in FACS‐sorted naïve (N), effector memory (EM), and central memory (CM) CD4^+^ and CD8^+^ T cells from spleen and lymph node pools of wild‐type mice (*n* = 3; data are presented as mean ± SD).

The miR‐17‐92 miRNAs have previously been implicated in T‐cell development and function, with reported expression in thymocytes and mature peripheral T cells [24, 29]. Consistent with observations in other cell lineages [22, 39], miR‐17‐92 expression progressively decreases as T‐cell differentiation proceeds, but remains expressed in mature T cells. Despite this, a consistent dataset capturing the expression of miR‐17‐92 miRNAs across defined T‐cell subsets, particularly in direct comparison to their shared pro‐apoptotic target gene *Bcl2l11*, hereafter referred to as *Bim*, has not been systematically reported. Hence, we performed qRT‐PCR on FACS‐sorted thymocyte subpopulations and peripheral T‐cell subsets. We quantified the three miR‐17‐92 family members for which we previously validated direct binding to the *Bim* 3′UTR [38]. In thymocytes, all three miRNAs were readily detected and showed a stage‐specific increase from the early developmental CD4^−^CD8^−^ double‐negative (DN) thymocyte subpopulations to more advanced CD4^+^CD8^+^ double‐positive (DP) thymocytes (Fig. 1A). In contrast, *Bim* mRNA levels increase less markedly over the same developmental trajectory (Fig. 1B), suggesting an inverse correlation between miR‐17‐92 and the *Bim* mRNA. In peripheral T cells, miR‐17‐5p and miR‐19b‐3p expression were decreased as compared to thymocytes and remained low across all subsets, while miR‐92a‐3p expression was not substantially downregulated and was finally moderately elevated in memory populations (Fig. 1C). Bim expression was highest in naïve T cells and reduced in memory subsets, particularly in CD8^+^ cells (Fig. 1D), suggesting an inverse relationship between miR‐92a‐3p and the *Bim* mRNA in CD8^+^ memory T cells. Together, these data confirm the developmental regulation of miR‐17‐92 cluster miRNAs in T cells and support a stage‐specific inverse relationship with *Bim* expression, which is most evident in the thymus.

### Early T‐cell development is not impacted upon loss of miR‐17‐92:Bim binding

**Fig. 2:**
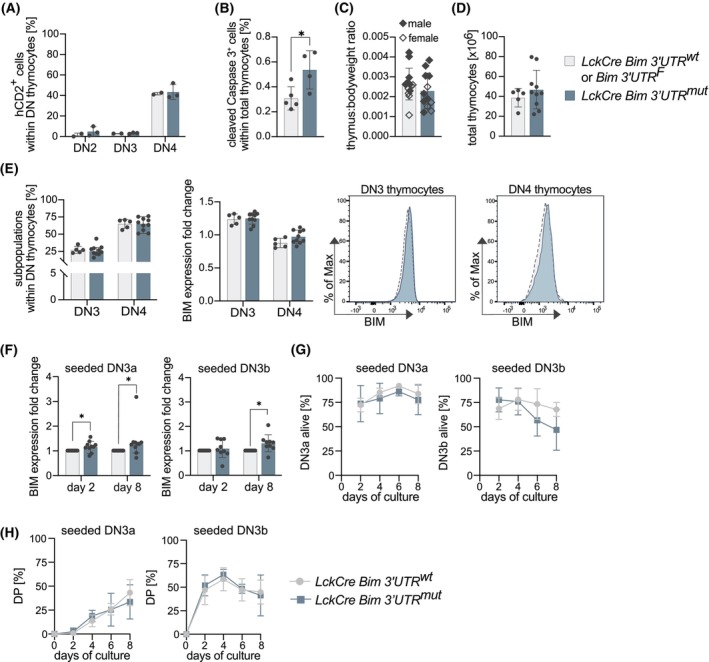
Early T‐cell development is not impacted upon loss of miR‐17‐92:Bim binding. (A) Cell surface expression of the human CD2 (hCD2) reporter, used as a proxy for Cre activity, on double‐negative (DN)2, DN3, and DN4 thymocytes of 8‐week‐old mice, analyzed by flow cytometry (LckCre Bim 3′UTR^wt^ (control): *n* = 2; LckCre Bim 3′UTR^mut^: *n* = 3; data are presented as mean ± SD). (B) The percentage of cleaved caspase 3^+^ thymocytes was determined by flow cytometry (control: *n* = 5; LckCre Bim 3′UTR^mut^: *n* = 4; statistical tests were performed using the unpaired Student's *t*‐test (*P < 0.05); data are presented as mean ± SD). (C) Thymus: body weight ratio of 8‐week‐old control and LckCre Bim 3′UTR^mut^ mice. No gender differences were observed (control: *n* = 13; LckCre Bim 3′UTR^mut^: *n* = 12; data are presented as mean ± SD). (D) Total thymocyte numbers of 8‐week‐old mice control and LckCre Bim 3′UTR^mut^ mice (control: *n* = 5; LckCre Bim 3′UTR^mut^: *n* = 10; data are presented as mean ± SD). (E) Fractions of double‐negative (DN)3 and DN4 populations within DN thymocytes and BIM expression in these subpopulations were analyzed by flow cytometry. BIM mean fluorescence intensity (MFI) of control and LckCre Bim 3′UTR^mut^ mice, both on a Ly5.2^+^ background, was normalized to BIM expression in wild‐type Ly5.1^+^ cells of the respective cell type. Ly5.1^+^ cells were spiked in the cell suspension and measured side by side in the same tube with the Ly5.2^+^ cells (control: *n* = 5; LckCre Bim 3′UTR^mut^: *n* = 10; data are presented as mean ± SD). (F) BIM protein expression was assessed by flow cytometry in FACS‐sorted double‐negative (DN)3a or DN3b thymocytes cocultured with OP9‐DL1 feeder cells to foster differentiation. Cells were analyzed on days 2 and 8 of culture. The mean fluorescence intensity (MFI) of BIM in LckCre Bim 3′UTR^mut^ cells was normalized to the respective MFI of control cells (control: double negative (DN)3a *n* = 10; DN3b *n* = 9; data are presented as median with 95% CI; LckCre Bim 3′UTR^mut^: DN3a *n* = 10; DN3b *n* = 9; data are presented as mean ± SD; statistical tests were performed using the unpaired Student's *t*‐test and Mann–Whitney U‐test (*P < 0.05)). (G) Fractions of viable cells in the cultures described in (F) (control: *n* = 5; LckCre Bim 3′UTR^mut^: *n* = 4; due to technical problems *n* = 4 for double negative (DN)3b on d 6; data are presented as median with 95% CI). (H) Fractions of double‐positive (DP) cells derived *in vitro* from FACS‐sorted and cultivated double negative (DN)3a and DN3b thymocytes as described in (F) (control: *n* = 5; LckCre Bim 3′UTR^mut^: *n* = 4; due to technical problems *n* = 4 for DN3b on d 6; data are presented as median with 95% CI).

To assess a role for direct repression of BIM protein levels by the miR‐17‐92 family miRNAs in T‐cell development, we crossed mice harboring a *lox*P‐flanked wild‐type *Bim* 3′UTR (*Bim 3*′*UTR* ^*F*^) [38] with mice expressing the Cre recombinase under control of the T‐cell‐specific Lck promoter (LckCre) [40], termed LckCre Bim 3′UTR^mut^ hereafter. Upon Cre‐mediated excision, which was reported to be initiated in the early developmental stage of CD4^−^CD8^−^ double‐negative (DN) 2 thymocytes [40], the wild‐type 3′UTR becomes replaced by a counterpart harboring mutations in all predicted binding sites for the miR‐17‐92 family miRNAs. In a previous study, we demonstrated that these mutations are functional and prevent physical binding of the miR‐17‐92 family miRNAs to the *Bim* mRNA 3′UTR [38]. To monitor the efficiency of the Cre‐mediated 3′UTR exchange and test whether Bim 3′UTR^mut^ thymocytes have a competitive disadvantage during thymocyte development, we made use of a Cre‐inducible hCD2‐reporter transgene encoded by one *R26* allele [41], which we had crossed to LckCre Bim 3′UTR^mut^ and control LckCre Bim 3′UTR^wt^ mice, respectively. Assessing hCD2 thymocyte surface expression by flow cytometry, we found the expected lack in early DN2 cells, and a gradual increase with developmental progression through the DN3 and DN4 subsets (Fig. 2A). However, LckCre Bim 3′UTR^mut^ DN thymocytes were not outcompeted by the remaining Bim 3′UTR^wt^ counterparts (Fig. 2A). Intriguingly, despite showing slightly elevated apoptosis rates in total thymocytes, as assessed by flow cytometry for cleaved Caspase 3 (Fig. 2B), we did not observe differences in thymus:body weight ratio (Fig. 2C) or total thymocyte counts (Fig. 2D) between the genotypes. Previous studies had reported roles for both, miR‐17‐92 family miRNAs and BIM, downstream of IL‐7R signaling and TCR ß‐selection in DN thymocytes, such as [2, 6, 30, 32]. However, we did not find any differences between the genotypes in the fractions at the DN3‐DN4 stages nor did we detect differences in BIM expression, as assessed by flow cytometry (Fig. 2E). Next, we FACS‐sorted DN3a (CD44^−^CD25^+^CD28^−^) and DN3b (CD44^−^CD25^+^CD28^+^) cells from both genotypes and cultivated these cells *in vitro* on OP9‐DL1 feeder cells [42] for 8 days. However, despite a tendency of increased BIM protein levels (Fig. 2F), neither the fraction of live cells (Fig. 2G) nor the developmental progression *in vitro* into the subsequent CD4^+^CD8^+^ double‐positive (DP) thymocyte subset differed between the genotypes (Fig. 2H). Therefore, impaired miR‐17‐92:Bim binding does not appear to affect cell survival at the different DN thymocyte stages.

### BIM expression is partially controlled by miR‐17‐92 in DP thymocytes and contributes to glucocorticoid‐induced apoptosis

**Fig. 3:**
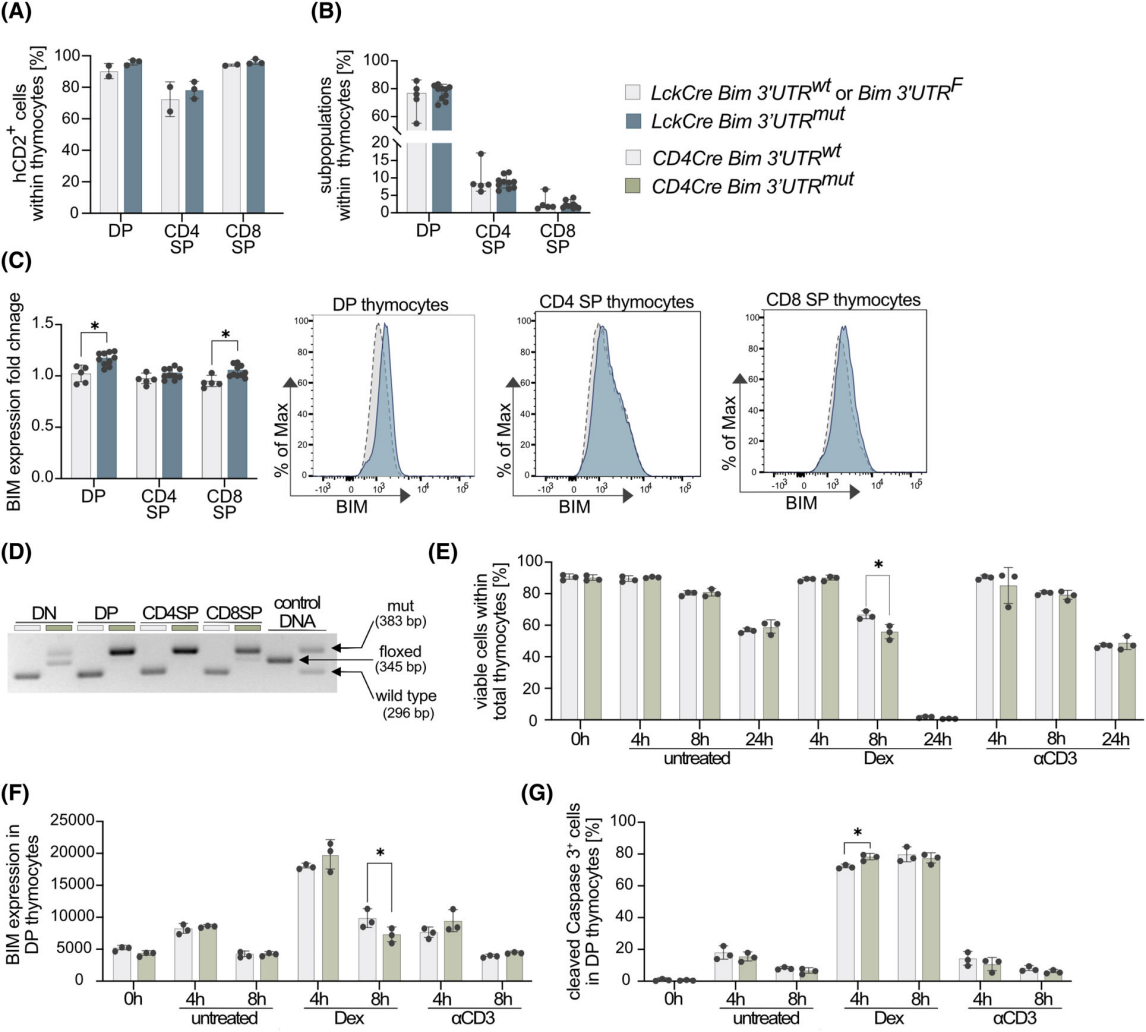
BIM expression is partially controlled by miR‐17‐92 in double‐positive (DP) thymocytes and contributes to glucocorticoid‐induced apoptosis. (A) Cell surface expression of the human CD2 (hCD2) reporter, used as a proxy for Cre activity, on double‐positive (DP), CD4 single positive (SP), and CD8 SP thymocytes of 8‐week‐old mice, analyzed by flow cytometry (LckCre Bim 3′UTR^wt^ (control): *n* = 2; LckCre Bim 3′UTR^mut^: *n* = 3; data are presented as mean ± SD). (B) Double positive (DP), CD4 single positive (SP), and CD8SP fractions within total thymocytes in 8‐week‐old control and LckCre Bim 3′UTR^mut^ mice were assessed by flow cytometry (control: *n* = 5; LckCre Bim 3′UTR^mut^: *n* = 10; data are presented as median with 95% CI). (C) BIM expression in double positive (DP), CD4 single positive (SP), and CD8 SP subpopulations was analyzed by flow cytometry. BIM MFI of control and LckCre Bim 3′UTR^mut^ mice, both on a Ly5.2^+^ background, was normalized to BIM expression in wild‐type Ly5.1^+^ cells of the respective cell type. Ly5.1^+^ cells were spiked in the cell suspension and measured side by side with the Ly5.2^+^ cells (control: *n* = 5; LckCre Bim 3′UTR^mut^: *n* = 10; data are presented as mean ± SD; statistical tests were performed using the unpaired Student's *t*‐test (* *P* < 0.05)). (D) Representative genotyping PCR of FACS‐sorted double negative (DN), double positive (DP), CD4 (single positive) SP, and CD8 SP thymocytes from CD4Cre Bim 3′UTR^wt^ and CD4Cre Bim 3′UTR^mut^ mice. (E–G) Total thymocytes from control and CD4Cre Bim 3′UTR^mut^ mice were treated with dexamethasone (Dex) or αCD3 for 4 h, 8 h and 24 h. At each time point, viability was assessed by flow cytometry; BIM expression and cleaved caspase 3^+^ cells were assessed by flow cytometry at 4 h and 8 h (control: *n* = 3; LckCre Bim 3′UTR^mut^: *n* = 3; data are presented as mean ± SD; statistical tests were performed using the unpaired Student's *t*‐test (*P < 0.05)).

We next assessed the DP as well as the further differentiated CD4^+^CD8^−^ and CD4^−^CD8^+^ single‐positive (SP) thymocyte subsets. In agreement with the findings in DN thymocytes, LckCre Bim 3′UTR^mut^ DP and SP thymocytes were not outcompeted by the remaining Bim 3′UTR^wt^ counterparts (Fig. 3A). Previous studies have demonstrated roles for BIM in promoting the apoptosis of DP thymocytes in response to developmental cues, such as lack of or autoreactive TCR signaling [2, 43, 44]. Although the composition of the DP and SP thymocyte subsets was not altered in LckCre Bim 3′UTR^mut^ mice (Fig. 3B), we did observe slightly elevated BIM levels in the mutant DP and CD8SP thymocytes, as assessed by flow cytometry (Fig. 3C). Thus, despite not affecting the size of the cell compartment at steady state, miR‐17‐92:Bim binding appears to be functional and relevant for BIM levels, particularly in DP thymocytes, potentially affecting their apoptotic priming. In addition to developmental cues, BIM has been reported to mediate apoptosis of DP thymocytes in response to exogenous cues, such as glucocorticoids [45, 46]. Further, glucocorticoid‐mediated repression of miR‐17‐92 contributes to apoptosis in various cell types [47, 48]. To exclude potential undetected phenotypic alterations during early T‐cell development, we established a second mouse strain for our investigations and crossed Bim 3′UTR mice with mice expressing the Cre recombinase under the control of the T‐cell‐specific CD4 promoter (CD4Cre) [40], termed CD4Cre Bim 3′UTR^mut^ hereafter. In these mice, Cre‐mediated exchange of the Bim 3′UTR is initiated in DP thymocytes, generating mice where all peripheral CD4^+^ and CD8^+^ T cells carry the mutated Bim 3′UTR. PCR confirmed efficient Cre‐mediated 3′UTR replacement in DP thymocytes (Fig. 3D). When we isolated thymocytes and treated the cells *in vitro* with an αCD3 to simulate negative selection‐like signals, we did not observe decreased cell viability, enhanced BIM expression or cleaved Caspase 3 signals in the mutant cells (Fig. 3E). Dexamethasone treatment *in vitro* slightly reduced cell viability after 8 h, decreased BIM levels after 8 h (Fig. 3F), and increased the fraction of cleaved Caspase 3^+^ apoptotic cells after 4 h (Fig. 3G). In summary, these results demonstrate that miR‐17‐92 may have minor roles in controlling BIM repression in thymocyte.

### Mature T‐cell homeostasis is unaffected in steady state by the loss of miR‐17‐92:Bim binding

**Fig. 4:**
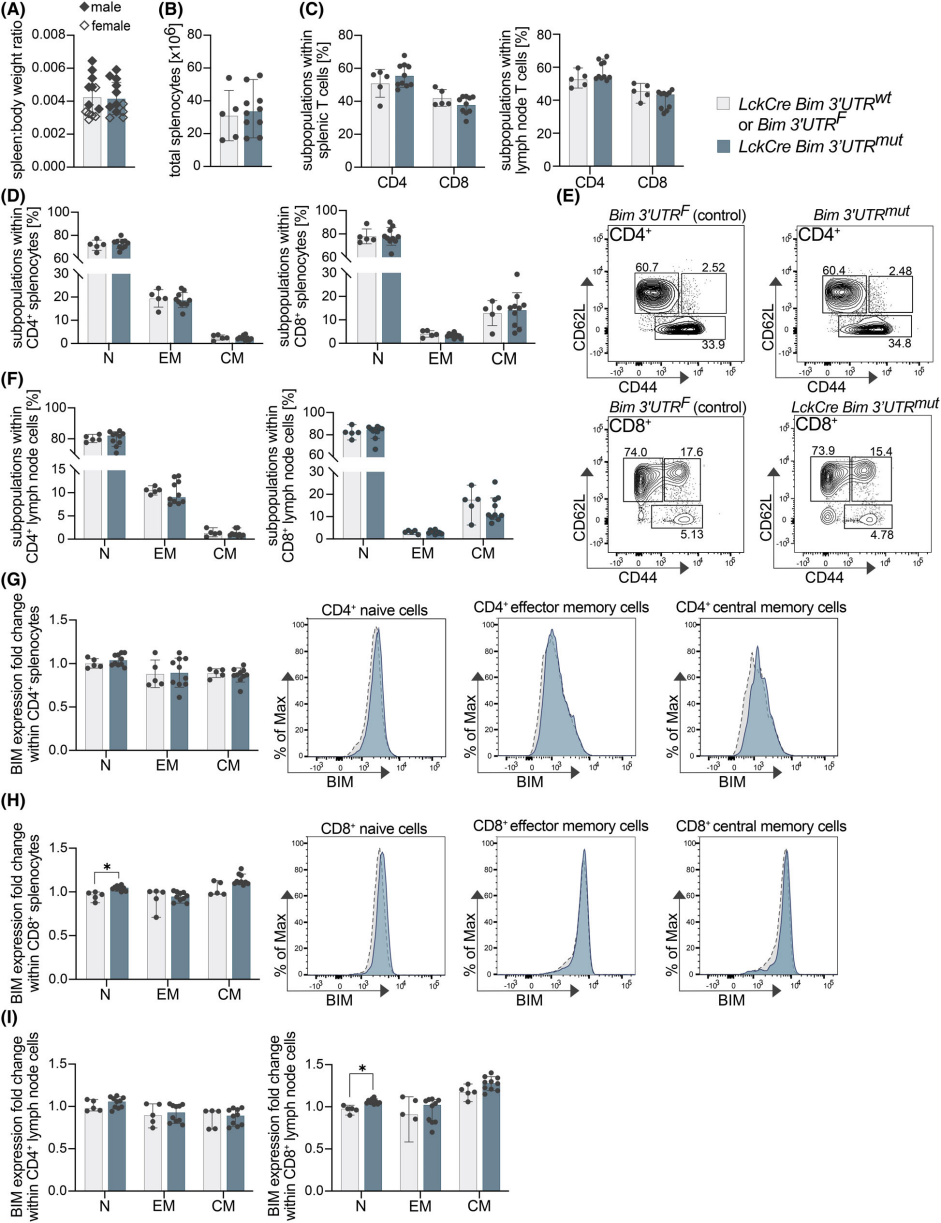
Mature T‐cell homeostasis is unaffected in steady state by the loss of miR‐17‐92:Bim binding. (A) Spleen: body weight ratio of 8‐week‐old control and LckCre Bim 3′UTR^mut^ mice (control: *n* = 13; LckCre Bim 3′UTR^mut^: *n* = 12; data are presented as mean ± SD). No gender differences were observed. (B) Total splenocyte count in 8‐week‐old control and LckCre Bim 3′UTR^mut^ mice (control: *n* = 5; LckCre Bim 3′UTR^mut^: *n* = 10; data are presented as mean ± SD). (C) CD4^+^ and CD8^+^ splenocytes and lymph node cells from 8‐week‐old control and LckCre Bim 3′UTR^mut^ mice (control: *n* = 5; LckCre Bim 3′UTR^mut^: *n* = 10); splenic T‐cell data are presented as mean ± SD and lymph node T‐cell data are presented as median with 95% CI. (D + E) Flow cytometry analysis of naïve (N), effector memory (EM) and central memory (CM) CD4^+^ and CD8^+^ splenocytes from 8‐week‐old control and LckCre Bim 3′UTR^mut^ mice are shown alongside a representative gating strategy (E) (control: *n* = 5; LckCre Bim 3′UTR^mut^: *n* = 10; data are presented as mean ± SD). (F) Proportions of naïve (N), effector memory (EM), and central memory (CM) CD4^+^ and CD8^+^ mesenteric lymph node cells from 8‐week‐old control and LckCre Bim 3′UTR^mut^ mice analyzed by flow cytometry (control: *n* = 5; LckCre Bim 3′UTR^mut^: *n* = 10; data are presented as median with 95% CI). (G–I) BIM protein levels in the indicated splenic or lymph node CD4^+^ and CD8^+^ subpopulations from 8‐week‐old control and LckCre Bim 3′UTR^mut^ mice, analyzed by flow cytometry (as described for Fig. 3C). Representative histograms of BIM mean fluorescence intensity (MFI) of CD8^+^ naive (N) and effector memory (EM) splenic T cells are shown. (control: *n* = 5; LckCre Bim 3′UTR^mut^: *n* = 10; data are presented as mean ± SD (G) or as median with 95% CI (H and I) statistical tests were performed using unpaired t‐test (G) or the Mann–Whitney U‐test (H and I) (*P < 0.05)).

Using the LckCre mouse model, we next investigated the functional importance of miR‐17‐92:Bim binding for the homeostasis of mature T cells in the spleen and mesenteric lymph nodes of 8‐week‐old mice. For the spleen, we did not observe differences in spleen:body weight ratio (Fig. 4A) or total cell counts between the genotypes (Fig. 4B). Further, LckCre Bim 3′UTR^mut^ mice did not differ from control mice in terms of fraction of CD4^+^ and CD8^+^ splenocytes and lymph node cells (Fig. 4C). Looking in more detail, we also could not find different fractions of naïve (N; CD62L^hi^CD44^lo^), effector memory (EM; CD62L^lo^CD44^hi^) and central memory (CM; CD62L^hi^CD44^hi^) CD4^+^ and CD8^+^ splenic (Fig. 4D,E) and mesenteric lymph node T cells (Fig. 4F), respectively. Whereas splenic CD4^+^ T cells did not show alterations in BIM expression (Fig. 4G), LckCre Bim 3′UTR^mut^ CD8^+^ naïve T cells in the spleen had slightly elevated BIM levels as compared to their LckCre Bim 3′UTR^wt^ controls (Fig. 4H). Of note, mesenteric lymph node T cells from LckCre Bim 3′UTR^mut^ mice showed a comparable effect (Fig. 4I). Similar to the analysis on thymocytes in these mice, and despite a slight increase in BIM levels in CD8^+^ naïve T cells, the bulk of naïve and antigen‐experienced CD4^+^ and CD8^+^ T cells appears unaffected during normal homeostasis when miR‐17‐92:Bim binding is absent.

### CD8 ^+^ memory formation is mildly affected by miR‐17‐92‐mediated suppression of BIM

**Fig. 5:**
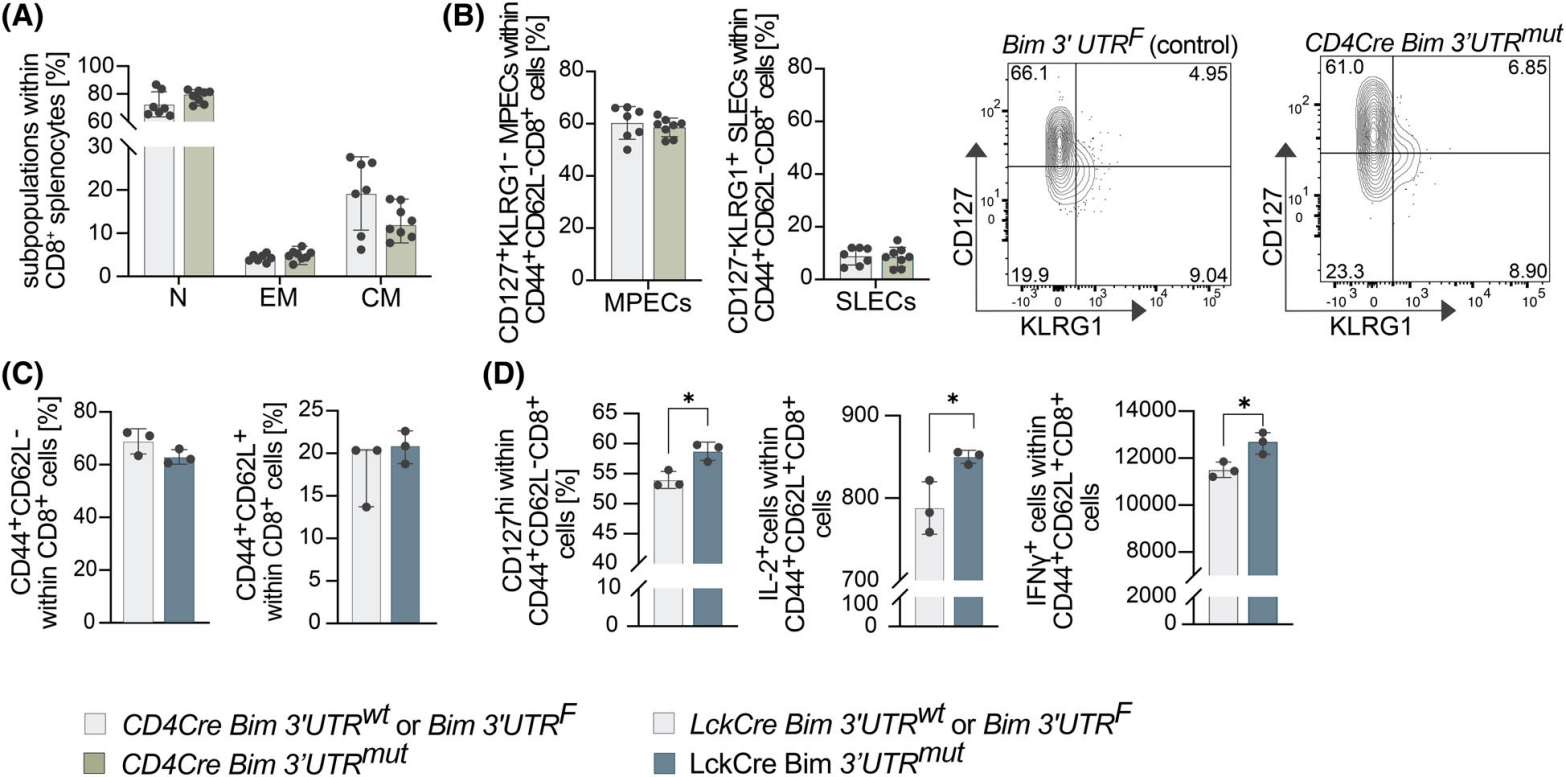
CD8^+^ memory formation is mildly affected by miR‐17‐92‐mediated suppression of BIM. (A) Proportions of naïve (N), effector memory (EM), and central memory (CM) cells within CD8^+^ splenocytes from 8‐week‐old CD4Cre Bim 3′UTR^wt^ (control) and CD4Cre Bim 3′UTR^mut^ mice were assessed by flow cytometry (control: *n* = 7; CD4Cre Bim 3′UTR^mut^: *n* = 8; data are presented as mean ± SD). (B) Flow cytometry analysis of CD127^+^KLRG1^−^ memory precursor effector cell (MPECs) within CD44^+^CD62L^−^CD8^+^ and CD127^−^KLRG1^+^ short‐lived effector cell (SLECs) within CD44^+^CD62L^−^CD8^+^ in spleens from 8‐week‐old control and CD4Cre Bim 3′UTR^mut^ mice are shown along a representative gating strategy (control: *n* = 7; CD4Cre Bim 3′UTR^mut^: *n* = 8; data are presented as mean ± SD). (C, D) CD8^+^ T cells isolated from splenocytes and lymph node cells of 8‐week‐old control and LckCre Bim 3′UTR^mut^ mice were cultured for 3 days in the presence of plate‐bound αCD3 and soluble αCD28. (C) Flow cytometry analysis was performed to assess the proportion of CD44^+^CD62L^−^ within CD8^+^ cells and CD44^+^CD62L^+^ within CD8^+^ cells in these cultures (control: *n* = 3; LckCre Bim 3′UTR^mut^: *n* = 3; data are presented as mean ± SD or median with 95% CI). (D) Within CD44^+^CD62L^−^CD8^+^ cells, the fraction of cells expressing CD127 was assessed and the expression of IL‐2 and IFNγ within CD44^+^CD62L^+^CD8^+^ cells was assessed (control: *n* = 3; LckCre Bim 3′UTR^mut^: *n* = 3; data are presented as mean ± SD; statistical tests were performed using the unpaired Student's *t*‐test (*P < 0.05)).

Previous reports had suggested a role for miR‐17‐92‐mediated suppression of BIM in promoting the survival of activated CD8^+^ T cells and their terminal differentiation into short‐lived effector cells (SLECs; CD127^−^KLRG1^+^) [33], which may occur at the expense of memory precursor effector cells (MPECs; CD127^+^KLRG1^−^). Thus, we aimed to further investigate the functional importance of miR‐17‐92:Bim interactions in CD8^+^ effector cells, using both our LckCre and CD4Cre mouse models. Similar to the LckCre model shown in Fig. 4, the fractions of naive CD8^+^ (N; CD62L^+^CD44^−^), effector memory CD8^+^ (EM; CD62L^−^CD44^+^), and central memory CD8^+^ (CM; CD62L^+^CD44^+^) subsets were comparable (Fig. 5A). Further, SLEC and MPEC fractions were comparable between CD4Cre Bim 3′UTR^mut^ and control mice (Fig. 5B). Next, we isolated CD8^+^ T cells from spleens and lymph nodes and cultured them under activating conditions. After 3 days of culture, approximately 70% of the viable CD8^+^ T cells had an effector memory‐like phenotype (EM; CD44^+^CD62L^−^), whereas approximately 20% of the cells had a central memory‐like phenotype (CM; CD44^+^CD62L^+^) (Fig. 5C). Surprisingly, LckCre Bim 3′UTR^mut^ cultures contained more cells expressing the memory marker CD127 and showed higher expression of IL‐2 and IFNγ as compared to control cultures (Fig. 5D). These results suggest that BIM silencing by miR‐17‐92 does not affect MPEC and SLEC formation at steady state. However, the *in vitro* culture analyses suggest that cell survival of CD8^+^ T cells upon activation may be impacted by miR‐17‐92‐mediated BIM repression.

### Loss of miR‐17‐92:Bim binding affects Th_17_ cell homeostasis

**Fig. 6:**
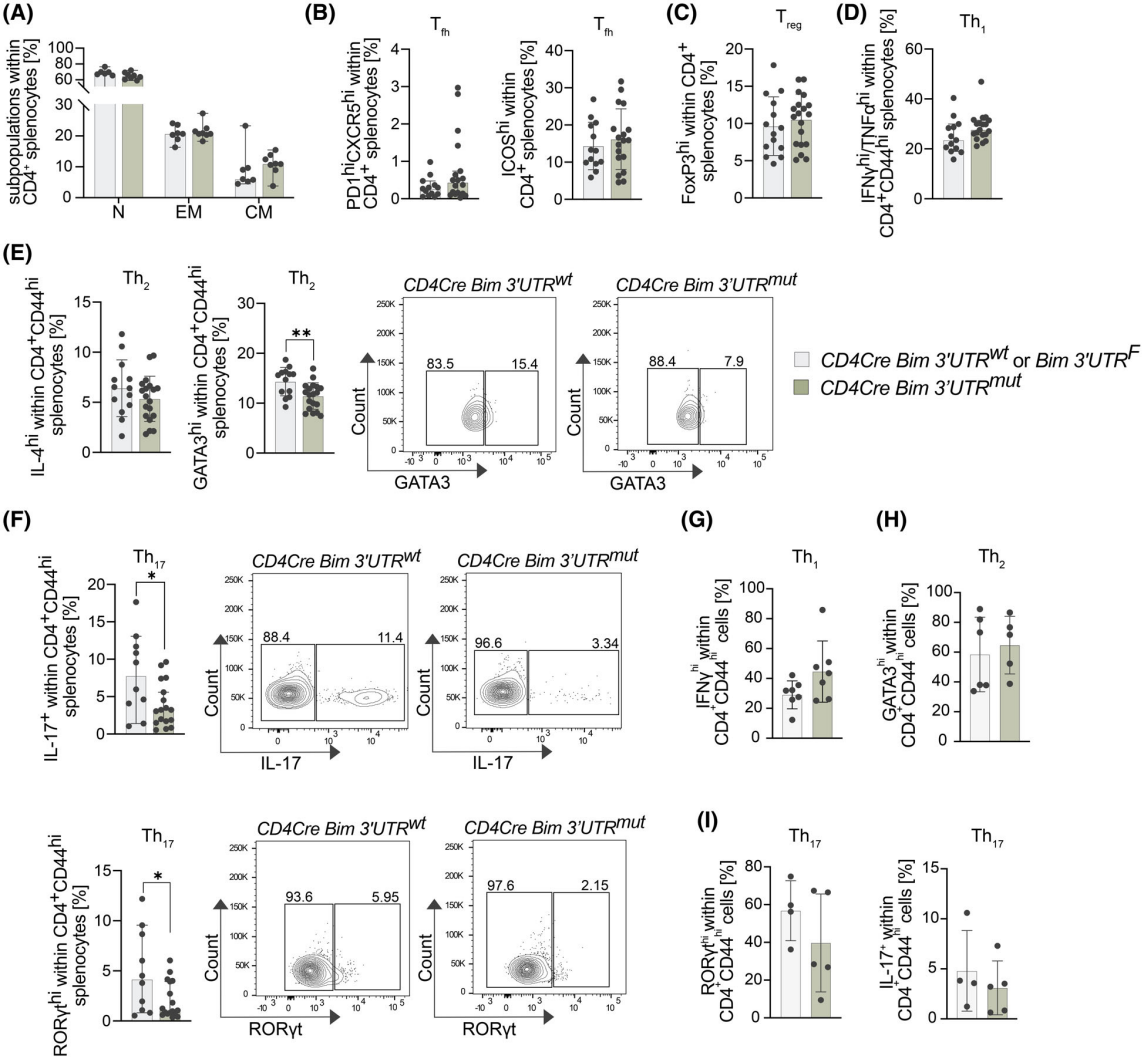
Loss of miR‐17‐92:Bim binding affects Th_17_ cell homeostasis. (A) Results from flow cytometry analysis of naïve (N), effector memory (EM), and central memory (CM) T cells within CD4^+^ splenocytes from 8‐week‐old control and CD4Cre Bim 3′UTR^mut^ mice (control: *n* = 7; CD4Cre Bim 3′UTR^mut^: *n* = 8; data are presented as mean ± SD). (B–F) Different CD4^+^ helper subsets in spleens of 8‐week‐old control and CD4Cre Bim 3′UTR^mut^ mice were assessed by flow cytometry. The bar graphs show fractions of (B) T_fh_ (PD1^hi^CXCR5^hi^ or ICOS^hi^; control: *n* = 13; CD4Cre Bim 3′UTR^mut^: *n* = 18; data are presented as median with 95% CI (PD1^hi^CXCR5^hi^) or mean ± SD (ICOS^hi^)) cells and (C) T_reg_ (FoxP3^hi^; control: *n* = 14; CD4Cre Bim 3′UTR^mut^: *n* = 19; data are presented as mean ± SD) cells within CD4^+^ splenocytes. Bar graphs in (D) show fractions of Th_1_ (IFNγ^hi^ TNFα^hi^; control: *n* = 13; CD4Cre Bim 3′UTR^mut^: *n* = 19; data are presented as median with 95% CI) cells within CD4^+^CD44^+^ splenocytes. Furthermore, the fractions of (E) Th_2_ (IL‐4^hi^ and GATA3^hi^; control: *n* = 13; CD4Cre Bim 3′UTR^mut^: *n* = 19; data are presented as mean ± SD) and (F) Th_17_ (IL‐17^+^ and RORγt^hi^; control: *n* = 10; CD4Cre Bim 3′UTR^mut^: *n* = 17; data are presented as median with 95% CI) cells within CD4^+^CD44^+^ splenocytes are shown. For (E) and (F), the gating strategy is shown for representative samples. (Statistical tests were performed using the unpaired Student's *t*‐test and Mann–Whitney U‐test (*P < 0.05; **P < 0.01). (G–I) *In vitro* differentiation assay of Th_1_, Th_2_, and Th_17_ subsets was performed for 3 days using isolated CD4^+^ T cells, and followed by flow cytometry analysis. The bar graphs show fractions of (G) Th_1_ cells (INFγ (control: *n* = 7; CD4Cre Bim 3′UTR^mut^: *n* = 7); data are presented as mean ± SD), Th_2_ cells (GATA3 (control: *n* = 6; CD4Cre Bim 3′UTR^mut^: *n* = 5); data are presented as mean ± SD), and (I) Th_17_ cells (RORγt or IL‐17 (control: *n* = 4; CD4Cre Bim 3′UTR^mut^: *n* = 5); data are presented as mean ± SD).

Finally, we analyzed the CD4^+^ T‐cell compartment in CD4Cre Bim 3′UTR^mut^ mice at steady state. Previous studies had reported roles for both BIM and miR‐17‐92 in different CD4^+^ effector subtypes [7, 12, 24]. Similar to the LckCre model shown in Fig. 4, the fractions and numbers of naïve, effector memory, and central memory CD4^+^ T cells were comparable between CD4Cre Bim 3′UTR^mut^ and control splenocytes (Fig. 6A). To identify CD4^+^ effector cell fates whose generation may be affected by direct binding of miR‐17‐92 to the *Bim* mRNA, we performed intracellular flow cytometry analyses for various signature transcription factors and cytokines in CD4^+^ splenocytes from 8‐week‐old mice. We could not detect differences in T_fh_, T_reg_, and Th_1_ cells (Fig. 6B–D). However, the fractions of Th_2_ cells were moderately decreased and the fractions of Th_17_ cells were significantly decreased (Fig. 6E,F). *In vitro* polarization experiments performed on isolated CD4^+^ cells revealed similar tendencies for Th_1_ and Th_17_ cells, although our data did not reach statistical significance (Fig. 6G–I). This result indicates that repression of BIM by miR‐17‐92 may serve the survival of Th_17_ cells, either during their generation or maintenance.

## Discussion

The miR‐17‐92 cluster and BIM play important roles in T‐cell development and homeostasis, shaping the naïve, effector, and memory cell pools. Both miR‐17‐92 and BIM are expressed across T‐cell subsets. Our data indicate that miR‐17‐92 expression decreases during T‐cell development, whereas *Bim* mRNA shows an inverse expression pattern, at least in thymocytes. Notably, while BIM has a well‐defined role in apoptosis [49], miR‐17‐92 has diverse roles in cell differentiation, proliferation, and apoptosis [50].

Interestingly, in mice both miR‐17‐92 overexpression and Bim deficiency lead to the aberrant survival of lymphocytes and autoimmunity [2, 24, 46, 51]. To study the specific relevance of BIM repression by miR‐17‐92, we generated a mouse model allowing the conditional joint ablation of all 9 putative miR‐17‐92 binding sites in the Bim 3′UTR [38]. Surprisingly, blocking miR‐17‐92:Bim interactions did not affect overall immune cell numbers but corrected the CD4^+^ and CD8^+^ T‐cell hyperplasia caused by *Bim* haploinsufficiency [38]. Thus, we explored whether miR‐17‐92‐mediated BIM repression may play a role in T‐cell development or polarization at steady state. To this aim, we generated mice in which miR‐17‐92:Bim binding was selectively blocked in T cells.

In these mice, T‐cell development was not phenotypically altered, and *in vitro* cultivated DN3a or DN3b cells efficiently differentiated into DP thymocytes. However, loss of miR‐17‐92:Bim binding mildly elevated BIM levels in DN3a and DN3b cells *in vitro*, and DP as well as CD8SP thymocytes *in vivo*. In addition to developmental cues, BIM mediates apoptosis of DP thymocytes in response to exogenous signals like glucocorticoids, whose repression of miR‐17‐92 also contributes to apoptosis in various cell types [45, 46, 47, 48]. Indeed, blocked miR‐17‐92:Bim binding caused a minor increase in cleaved Caspase3^+^ cells *in vitro* upon dexamethasone. Thus, miR‐17‐92‐mediated repression of BIM protein expression appears to play a limited role in protecting thymocytes from apoptosis. Further mRNA targets of miR‐17‐92 may serve anti‐apoptotic functions. PTEN, the major negative regulator of PI3K signaling which promotes DN thymocyte survival downstream of IL‐7R and pre‐TCR signaling [52] is another validated direct joint miR‐17‐92 target, with at least 8 putative binding sites [24]. PI3K signaling negatively affects BIM levels via AKT‐ or ERK1/2‐mediated BIM degradation or transcriptional repression via FOXO3a, all of which represent indirect modes of regulation of BIM levels via miR‐17‐92 [53]. Of note, PTEN has been established as a miR‐17‐92 target in thymocytes [30, 32].

In line with unaltered thymocyte numbers, peripheral CD4^+^ and CD8^+^ T‐cell numbers are not affected upon loss of miR‐17‐92:Bim binding. Although the lifespan of at least naïve CD4^+^ cells is restricted partially by BIM‐mediated apoptosis [8], BIM regulation in these cells seems independent of miR‐17‐92. CD8^+^ naïve T cells had slightly elevated BIM levels when miR‐17‐92:Bim binding was inhibited; however, despite the accumulation of both CD4^+^ and CD8^+^ naïve T cells in *Bim* knockout mice, little is known about CD8^+^ T cells in this context. Of note, the lifespan of naïve T cells extends during organismal aging [54], and whether dampening BIM levels by miR‐17‐92 could be involved in this phenomenon remains an open question.

Although both, BIM and miR‐17‐92 affect the polarization or functionality of most CD4^+^ helper T‐cell subsets, loss of miR‐17‐92:Bim binding leads to a specific decrease in the fractions of Th_17_ cells. For T_fh_ cells, this is somewhat surprising as aberrant miR‐17‐92 expression causes autoimmunity that critically involves T_fh_ cells [24, 29], and BIM‐deficient mice are prone to the same autoimmune disease, that is SLE [46]. Further, prominent roles for miR‐17‐92 in promoting T_fh_ cell polarization have been described [28, 29]. However, PTEN and the PI3K‐inhibiting phosphatase PHLPP2 appear to be the critical targets in this context [28]. Conversely, although roles for miR‐17‐92 in Th_17_ polarization have been described (reviewed in [31]), reports on apoptosis in Th_17_ biology are sparse. Thus, we speculate that repression of BIM by miR‐17‐92 may inhibit apoptosis during Th_17_ polarization or maintenance of the Th_17_ pool, a hypothesis that calls for further investigation. For Th_2_ cells, BIM‐mediated apoptosis was described to maintain the cell pool during airway inflammation in the lung [55], whereas the limited reports for miR‐17‐92 may suggest roles in restraining Th_2_ cell function [56]. However, our data suggest no major roles for miR‐17‐92:Bim binding in Th_2_ cells at steady state. Finally, miR‐17‐92 has been proposed to exert vigorous control over the Th_1_/T_reg_ balance [36], a function that appears to be independent of the target *Bim* mRNA, as we do not observe altered fractions or a relative shift in these T‐cell subsets.

In activated CD8^+^ T cells, miR‐17‐92 promotes a short‐lived effector cell fate, while downregulation of miR‐17‐92 fosters a memory fate [33, 34]. Conversely, CD8^+^ effector cells with high BIM and along high BCL2 levels have a greater memory potential [11, 16]. However, our results show that in steady state *in vivo*, miR‐17‐92 downregulation is unlikely to be responsible for the substantial BIM upregulation in CD8^+^ cells that acquire a memory fate. Nevertheless, *ex vivo* activated CD8^+^ T cells with memory surface markers express more IL‐2 and IFNγ, which may indicate a selective advantage of such cells. Potentially higher BIM levels could enforce increased expression of the pro‐survival BCL‐2 protein, a scenario that has been described in memory‐prone CD8^+^ T cells *in vivo* [11, 16]. Therefore, repression of BIM by miR‐17‐92 may tilt the balance between effector and memory cells towards effector cell fates. This could have implications for the outcome of CD8^+^ T‐cell responses upon vaccination, infections, but also tumor cells, all of which should be tested in the future.

In conclusion, our study reveals that direct repression of BIM by miR17‐92 is of limited importance for the establishment and maintenance of a healthy T‐cell pool in unchallenged mice. However, our findings suggest specific roles for BIM repression by miR‐17‐92, such as in establishing and/or maintaining the Th_17_ cell pool.

## Materials and methods

Chemicals and antibodies used are listed in the Tables S1–S3.

### Mice

All mice have been generated on the C57BL/6 background or have been backcrossed to the C57BL/6 background for more than 10 generations. LckCre [40], CD4Cre [40], R26hCD2^stopF^ [41], and CD45.1 (Ly5.1) [57] were kindly provided by Prof. Andreas Villunger. Bim 3′UTR^mut^ mice were described in our initial report on the generation of this mouse line [38]. All mice were bred and maintained at the central laboratory animal facility of the Medical University of Innsbruck under controlled conditions (temperature 22 ± 2 °C, relative humidity of 55–65%, 12‐h light/dark cycle). No experimental procedures were performed. Animals were euthanized at 8 weeks of age, and lymphatic organs were harvested. Animals did not suffer unnecessarily at any stage of their life. Both sexes were used for experiments, and no differences between male and female mice were found in the parameters analyzed. Animal care and experiments were approved by the Austrian Ministry of Education, Science and Research (BMWF‐66. 66.011/0017‐II/3b/2014).

### Preparation of single‐cell suspensions

For all preparation, washing and staining procedures, FACS‐buffer [PBS, 3% FBS, 50 μL·mL^−1^ Gentamicin, 100 U·mL^−1^ Penicillin/100 μg·mL^−1^ Streptomycin] was used. Washing was performed at 1500 rpm for 5 min at 4 °C. Single‐cell suspensions were prepared by mechanical disruption of thymus, spleen or a mixture of superficial cervical, axillary, brachial, inguinal, and mesenteric lymph nodes through 70 μm mesh filters in FACS‐buffer. Cells were washed, pellets were resuspended in FACS‐buffer and filtered through a 50 μm filter. For erythrocyte lysis of spleen, cells were washed, each pellet was resuspended in 1 mL ice‐cold lysis buffer [155 mm NH_4_Cl, 10 mm KHCO_3_, 0.1 mm EDTA; pH 7.5] and left on ice for 3 min. About 3 mL FACS‐buffer were added, cells were washed, resuspended in FACS‐buffer and filtered through a 50 μm filter. Cell number was determined using a hemocytometer and trypan blue exclusion.

### TaqMan‐based expression analysis of miR‐17‐5p, miR‐19b‐3p and miR‐92a‐3p in T‐cell subsets

Subpopulations of thymocytes and T cells derived from pooled spleen and lymph node cells were FACS‐sorted. For total RNA isolation, the miRNeasy Mini Kit (Qiagen, Venlo, The Netherlands; 217 004) was used. miR‐17‐5p, miR‐19b‐3p, and miR‐92a‐3p were reverse transcribed using the TaqMan MicroRNA Reverse Transcription Kit (Thermo Fisher Scientific, Waltham, MA, USA; 4 366 596). Real‐time qRT‐PCR was performed using the TaqMan™ Fast Advanced Master Mix for qPCR (Thermo Fisher Scientific, 4 444 556) with the following primers (Thermo Fisher Scientific, 4 427 975): hsa‐miR‐17 (Assay ID: 002308), hsa‐miR‐19b (Assay ID: 000396), hsa‐miR‐92 (Assay ID: 000430), and U6 snRNA (Assay ID: 001973).

### RT‐qPCR for *Bim* and *Hprt*

Subpopulations of thymocytes and T cells derived from pooled spleen and lymph node cells were FACS‐sorted. For total RNA isolation, the Quick‐RNA™ MicroPrep Kit was used according to the manufacturer's instructions. cDNA synthesis from total RNA was performed using the iScript cDNA Synthesis Kit, and the Universal qPCR Master Mix was used for RT‐qPCR analyses with the following primers: *Hprt* sense: GTCATGCCGACCCGCAGTC, *Hprt* antisense: GTCCTTCCATAATAGTCCATGAGGAATA AAC, *Bim* sense: GAGATACGGATTGCACAGGA, *Bim* antisense: TCAGCCTCGCGGTAATCAT.

### Cell culture

All cells were cultured at 37 °C with 5% CO_2_. OP9‐DL1 cells (OP9/G‐DLL1, RRID: CVCL_B218; murine OP9 bone marrow stromal cell line stably expressing Delta‐like 1) were kindly provided by Prof. Andreas Villunger. Mycoplasma‐free cells were used for all experiments. OP9‐DL1/thymocyte cocultures were performed as described [42]. Briefly, OP9‐DL1 cells were cultured in RPMI‐1640 medium containing 20% FBS, 2 mm l ‐glutamine, 100 U·mL^−1^ penicillin/100 g·mL^−1^ streptomycin, 1% nonessential amino acids, 1 mm sodium pyruvate and 55 μm b‐Mercaptoethanol. 5 × 10^4^ OP9‐DL1 cells/well were seeded in flat‐bottom 96‐well plates. After 24 h, 10 μg·mL^−1^ Mitomycin C was added for 2 h. OP9‐DL1 cells were washed 5× with PBS before seeding 5 × 10^3^ FACS‐sorted double‐negative (DN3a, DN3b) thymocytes/well on top. Cocultures were performed in MEMα containing 10% FBS, 100 U·mL^−1^ penicillin/100 μg·mL^−1^ streptomycin, 55 μm b‐Mercaptoethanol, 50 μL·mL^−1^ Gentamicin, 1 ng·mL^−1^ IL‐7 and 5 ng·mL^−1^ FLT3‐Ligand. Cells were cocultured for 8 days. At day 4, the medium was renewed. For CD8^+^ T‐cell activation, 24‐well plates were coated with αCD3 (2 μg·mL^−1^ in 500 μL PBS (pH 7.4)/well) for 24 h at 4 °C. CD8^+^ T cells were isolated from pooled spleen and lymph nodes (pool of mesenteric, inguinal, axillary, and brachial lymph nodes) as per manufacturer's instructions (Miltenyi Biotec). 2 × 10^6^ CD8^+^ T cells were plated in previously αCD3‐coated wells and cultured in the presence of 0.5 μg·mL^−1^ αCD28 in IMDM containing 10% FBS, 2 mm l ‐glutamine, and 100 U·mL^−1^ penicillin/100 μg·mL^−1^ streptomycin.

### CD4 ^+^ T‐cell isolation and *in vitro* stimulation

Unless otherwise stated, all centrifugation steps were performed at 1500 rpm for 5 min at 4 °C. Mixtures of cells originating from the spleen, superficial cervical, axillary, brachial, inguinal, and mesenteric lymph nodes were used to prepare single‐cell suspensions. Each cell suspension was incubated with 300 μL of staining cocktail for 15 min on ice in FACS buffer, containing biotin‐labeled antibodies against CD11b, Ter119, NK1.1, Gr.1, CD8, B220, and CD19. Single‐cell suspensions were centrifuged, transferred into plastic tubes (Falcon™, 352 054), and resuspended in 100 μL MACS C buffer (PBS, 0.5% BSA, 2 mm EDTA) and 5 μL MagniSort Streptavidin Negative Selections Beads per 1 × 10^7^ cells. The mixture was incubated for 5 min at room temperature. The volume was brought to 3 mL, the tubes were placed for 10 min at room temperature on a cell separation magnet. The suspension was transferred into fresh tubes, and the separation step was repeated. Single‐cell suspensions were centrifuged, resuspended in complete IMDM medium supplemented with 10% FBS, 1% l‐glutamine, and 100 U·mL^−1^ penicillin/100 μg·mL^−1^ streptomycin. Cell number was determined using the trypan blue exclusion method. For each *in vitro* polarization sample, 5 × 10^5^ isolated CD4^+^ T cells were used. Cytokine cocktails were prepared for 200 μL per polarization condition in IMDM medium (as described above), and cells were seeded in a 96‐well flat‐bottom plate. For all conditions cells were cultured in the presence of plate bound αCD3 and soluble αCD28 (5 μg·mL^−1^). For plate bound αCD3, 2 μg·mL^−1^ αCD3 in 200 μL Tris Buffer (50 mm, pH 9.0) was incubated for at least 12 h at 4 °C, and the wells were washed 2× with PBS before seeding cells. Additionally, their corresponding polarizing cytokine and antibody mixes in IMDM medium was added. For Th_1_ polarization, cells were cultured with αIL‐4 (0.5 μg·mL^−1^) and IL‐12 (10 ng·mL^−1^). For Th_2_ polarization, cells were treated with IL‐4 (10 ng·mL^−1^), αIL‐12 (5 μg·mL^−1^), and αIFNγ (5 μg·mL^−1^). For Th_17_ polarization, cells were stimulated with IL‐23 (10 ng·mL^−1^), TGF‐β (5 ng·mL^−1^), IL‐6 (20 ng·mL^−1^), αIL‐4 (5 μg·mL^−1^), and αIFNγ (5 μg·mL^−1^). On day 3 of culture, cells were transferred to 96‐well U‐bottom plates by vigorous pipetting, and intracellular cytokines as well as transcription factor expression were analyzed by flow cytometry.

### *In vitro* apoptosis assay of thymocytes

2 × 10^5^ thymocyte suspension per well of a 96‐well plate were used for each condition. Plate bound αCD3 (2 μg·mL^−1^) was prepared as described for the CD4^+^ T‐cell isolation and *in vitro* stimulation. Dexamethasone was added at a concentration of 10^−7^ m. Treated and untreated thymocytes were cultured in IMDM medium supplemented with 10% FBS, 1% l‐glutamine, and 100 U·mL^−1^ penicillin/100 μg·mL^−1^ streptomycin. After 4 h, 8 h, and 24 h, the cells were harvested and stained for viable cells (Fixable Viability Dye), cleaved Caspase 3 and BIM by flow cytometry.

### Flow cytometry and cell sorting

For all washing steps, unless otherwise stated, 150 μL cold FACS‐buffer was used and cells were centrifuged at 2000 rpm for 2 min at 4 °C. All incubation steps were performed at 4 °C. For flow cytometry, up to 1 × 10^6^ cells were used per staining which was performed in 96‐U‐well plates. For cell sorting, entire organ single‐cell suspensions were used, and staining was performed in 2 mL reaction tubes.

All flow cytometry data were acquired using LSR Fortessa Cell Analyzer (BD Bioscience, Franklin Lakes, NJ, USA) and the FACSDiva software (BD Bioscience, Version 9.0.1). Cell sorting was performed using a FACSAria III cell sorter (BD Bioscience) and the FACSDiva software (BD Bioscience, Version 9.0.1). Data were analyzed using Flowjo software (Tree Star, Ashland, OR, USA, Version 10). FSC‐H/FSC‐W and SCC‐H/SSC‐W features were used to exclude nonsinglet events.

### Cell surface staining for flow cytometry

Cell pellets were resuspended and incubated in 25 μL TruStain FcX™ (1:200 in FACS‐buffer) for 10 min. About 25 μL antibody cocktail was added on top, and the cells were incubated for 20 min. Cells stained with biotinylated antibodies were washed twice, resuspended in 30 μL of fluorochrome‐conjugated streptavidin in FACS‐buffer, and incubated for 15 min. Cells were washed three times with PBS and incubated in Fixable Viability Dye (1:2000 in PBS) for 10 min. Afterwards, cells were washed three times, resuspended in 120 μL FACS‐buffer, and transferred to micro‐titer tubes for measurement.

### Intracellular cytokine and transcription factor staining for flow cytometry

Cytokine production was determined after T‐cell restimulation for 4 h at 37 °C with PMA (50 ng·mL^−1^) and Ionomycin (500 ng·mL^−1^) in the presence of Monensin prior to staining. After cell surface staining, cells were fixed and permeabilized using the FoxP3 transcription factor kit according to the manufacturer's instructions (eBioscience, San Diego, CA, USA). Next, cells were incubated in 25 μL TruStain FcX™ (1:20 in FACS‐buffer) for 20 min, and 25 μL intracellular antibody cocktail was added, and cells were incubated for 20 min. Afterwards, cells were washed three times, resuspended in 120 μL FACS‐buffer, and transferred to micro‐titer tubes for measurement.

### Intracellular BIM and cleaved caspase 3 staining for flow cytometry

After cell surface staining, cells were fixed with Cytofix™ Fixation Buffer and permeabilized with Perm/Wash™ Buffer according to the manufacturer's instructions (BD Bioscience). Next, cells were resuspended in 25 μL TruStain FcX™ (1:20 in FACS‐buffer) and incubated for 20 min. About 25 μL unlabeled αBIM antibody (1:200 in Perm/Wash™ Buffer) and/or PE‐labeled cleaved caspase 3 antibody (1:200 in Perm/Wash™ Buffer) was added, and cells were further incubated for 30 min. Cells were washed twice with Perm/Wash™ Buffer and centrifuged for 2 min at 2400 rpm at 4 °C, and incubated with 25 μL A488‐conjugated αIgG antibody (1:1000 in Perm/Wash™ Buffer) for 20 min to visualize bound BIM antibody. Cells were washed three times, resuspended in 120 μL FACS‐buffer and transferred to micro‐titer tubes for measurement. To detect minor differences in BIM levels in *ex vivo* analyses, as apoptotic cells are quickly phagocytozed *in vivo*, wild‐type or mutant cells were normalized to control cells that were measured side by side in the same tube. Thus, control cells derived from Ly5.1^+^ wild‐type mice were mixed 1:1 with Ly5.2^+^ wild‐type control or Bim 3′UTR mutant cells before staining procedures. Staining cocktails included antibodies for Ly5.1^+^ and Ly5.2^+^ to allow separate analysis of MFI and subsequent paired normalization (Figs 2F, 3C, 4G,H,I). For *in vitro* experiments, the MFI of the BIM staining was depicted either directly (Fig. 3F) or shown normalized to control cells in other wells (Fig. 2G).

### Cell sorting

Cell suspensions were incubated in 300 μL antibody cocktail in FACS‐buffer for 20 min. Samples were washed twice in 1 mL FACS‐buffer, centrifuged at 1500 rpm for 5 min at 4 °C. If biotinylated antibodies were used, cells were incubated for 15 min in 300 μL diluted fluorochrome‐conjugated streptavidin and washed three times in 1 mL FACS‐buffer. Cells were filtered through a 50 μm filter prior to sorting.

### Quantification and statistical analyses

Results are presented as mean ± standard deviation (SD) for normally distributed data, or as median with 95% confidence interval (CI) for non‐normally distributed data. Normality was assessed using the Shapiro–Wilk test (α = 0.05). Statistical differences between the Control and Mutant groups were evaluated for each cell subset using the unpaired t‐test for normally distributed data or the Mann–Whitney U‐test for non‐normally distributed data. The number of biological replicates (*n*) is provided in each figure legend. Data visualization and statistical analysis were performed using GraphPad Prism (Version 10.3.0). Figures were generated using Affinity Designer (Version 1.8.6) or BioRender.com. Asterisks (*P < 0.05, **P < 0.01, ***P < 0.001) indicate statistically significant differences.

## Conflict of interest

The authors declare no conflict of interest.

## Author contributions

VL conceived the study. KH, JW, JB, SS, NK, and WO designed, performed, and analyzed experiments. VL, KH, NK, JW, and AV interpreted the data. KH and VL wrote the manuscript. KH designed the figures. All authors contributed to the article and approved the submitted version.

## Supporting information


**Table S1.** Materials & chemicals.
**Table S2.** Antibodies for flow cytometry.
**Table S3.** Antibodies and stimulating components for CD4+ T‐cell isolation and T helper differentiation.

## Data Availability

The data that support the findings of this study are available on Zenodo at: https://doi.org/10.5281/zenodo.17483376.
