# Supplementary material for: Limited roles of the miR‐17‐92 cluster in the regulation of T‐cell apoptosis
Source: FEBS J. 2026 Jan 6;293(10):2845–61. doi: 10.1111/febs.70387 (PMC13193475; doi:10.1111/febs.70387)
Supplement: Supplementary file 1 — Table S1. Materials & chemicals. Table S2. Antibodies for flow cytometry. Table S3. Antibodies and stimulating components for CD4+ T‐cell isolation and T helper differentiation. [file FEBS-293-2845-s001.pdf]

Supporting information

Table S1: Materials & Chemicals

	Supplier	Identifier
FBS	Gibco	10270106
Gentamicin	Gibco	15750037
Penicillin/Streptomycin	Sigma	P0781
Trypan blue	VWR	97063-702
RPMI-1640	Sigma	R0883
L-Glutamine	Sigma	G7513
Sodium Pyruvate (100mM)	Gibco	11360070
Non-Essential Amino Acids	Gibco	11140035
b-Mercaptoethanol	Sigma	M3148
Mitomycin C	Merck	M0503
MEMα	Gibco	12000014
IL-7	Peprotech	217-17
FLT3-Ligand	Peprotech	250-31L
CD8a^{+} T-cell isolation kit	Miltenyi Biotec	130-104-075
αCD3	BioXcell	BE0001-1
αCD28	BioXcell	BE0015-1
IMDM	Sigma	I3390
TruStain FcX™(αCD16/32)	Biolegend	101320
FoxP3 transcription factor kit	eBioscience	00-5523-00
Cytofix™ Fixation Buffer	BD Bioscience	554655
Perm/Wash Buffer	BD Bioscience	554723
70 µm mesh filters	Falcon	352350
50 µm cup filcon	BD Bioscience	340632
micro-titer tubes	Biozym	710410N
Round-Bottom Polystyrene Tubes	Falcon	352054
Monensin Solution	Biolegend	420701
Ionomycin calcium salt	Sigma	10634
PMA	Sigma	P1269l
Dexamethasone	Sigma	D4902
BSA	Merck Millipore	12659
MagniSort Streptavidin Negative Selections Beads	ThermoFischer	MSNB-6002-74
Magnet BD IMag	BD Bioscience	552311
Quick-RNA™ MicroPrep	Zymo Research	R1050
iScript cDNA Synthesis Kit	BIO RAD	#1708891
Luna Universal qPCR Master Mix	New England BioLabs	M3003E

Table S2: Antibodies for flow cytometry.

**Antibodies for cell surface staining**				
Antibody	Clone	Dilution	Supplier	Identifier
αCD8-PE/Cy7	53-6.7	1:300	Biolegend	100722
αCD8-PerCP/Cy5.5	53-6.7	1:300	Biolegend	100734
αCD8-BV421	53-6.7	1:300	Biolegend	100738
αCD62L-PerCP/Cy5.5	MEL-14	1:300	Biolegend	104431
αCD4-PerCP/Cy5.5	RM4-5	1:200	eBioscience	45-0042-82
αCD4-PE	RM4-4	1:300	Biolegend	116006
αCD4-A700	RM4-4	1:300	Biolegend	116022

αCD4-FITC	GK1.5	1:200	eBioscience	11-0041-85
αCD3-A700	500A2	1:200	Biolegend	152316
αTCRß-PerCP/Cy5.5	H57-597	1:300	Biolegend	109228
αTCRß-FITC	H57-597	1:300	eBioscience	11-5961-85
αhCD2-BV421	TS1/8	1:300	Biolegend	309218
αhCD2-PE/Cy7	TS1/8	1:300	Biolegend	309214
αCD44-BV510	IM7	1:300	Biolegend	103044
αCD44-BV605	IM7	1:300	Biolegend	103047
αCD127 (IL-7Rα)-PE/Cy7	A7R34	1:300	Biolegend	135013
αNK1.1-FITC	PK136	1:300	Biolegend	108705
αNK1.1-PE/Cy7	PK136	1:400	Biolegend	108713
αKLRG1(MAFA)-APC	2F1/KLRG1	1:100	Biolegend	138411
αCD25-APC	PC61	1:300	Biolegend	102012
αCD25-PE	3C7	1:300	Biolegend	101904
αCD19-biotin	eBio1D3	1:300	eBioscience	13-0193-82
αCD11b-biotin	M1/70	1:300	Biolegend	101204
αGr1-biotin	RB6-8C5	1:300	Biolegend	108404
αTer119-biotin	Ter119	1:300	Biolegend	116204
αNK-1.1-biotin	PK136	1:200	Biolegend	108704
αCD45R(B220)-biotin	RA3-6B2	1:300	Biolegend	103204
αCD185(CXCR5)-biotin	L138D7	1:300	Biolegend	145510
αCD28-PE	37.51	1:300	Biolegend	102105
αCD279 (PD-1)-PE	RMP1-14	1:100	Biolegend	114117
αCD45-PE/Cy7	30-F11	1:600	Biolegend	103113
αCD117(cKit)-PerCP/Cy5.5	2B8	1:300	Biolegend	105823
CD45.1-PE	A20	1:200	eBioscience	12-0453-83
CD45.2-eFluor™ 506	104	1:100	eBioscience	69-0454-80
Fixable Viability Dye		1:2000	eBioscience	65-0866-14
**Strepdavidin-conjugates**				
Strep-PerCP/Cy5.5		1:200	eBioscience	45-4317-82
Strep-BV605		1:200	Biolegend	405229
**Antibodies for intracellular staining**				
αIFNγ-PE	XMG1.2	1:200	Biolegend	505808
αIFNγ-PE/Cy7	XMG1.2	1:200	Biolegend	505825
αRORγt-PE	AFKJS-9	1:300	eBioscience	12-6988-82
αCD278(ICOS)-PE/Cy7	7E.17G9	1:200	Biolegend	117421
αT-BET-APC	eBio4B10	1:200	eBioscience	50-5825-82
αIL-4-APC	11B11	1:200	Biolegend	504105
αIL-17-Alexa Fluor 647	TC11-15H10.1	1:200	Biolegend	506911
αFoxP3-APC	FJK-16s	1:200	eBioscience	50-5773-80
αTNFα-PerCP/Cy5.5	MP6-XT22	1:200	BD Bioscience	560659
αGATA3-eFluor™710	TWAJ	1:200	eBioscience	46-9966-42
αIL-2-BV421	JES6-5H4	1:200	Biolegend	503825
αIL-2-APC	JES6-5H4	1:200	Biolegend	503809
αBCL6-BV421	K112-91	1:200	BD Bioscience	563363
αEOMES-PE/Cy7	Dan11mag	1:200	eBioscience	25-4875-80
αBIM monoclonal	Y36	1:200	Abcam	ab32158
Cleaved Caspase-3-PE	C92-605	1:300	BD Bioscience	570183
IgG (H+L) Secondary Antibody-A488		1:1000	Invitrogen	A-11034
**Antibodies used for cell sorting**				

αCD28-PE	37.51	1:100	Biolegend	102105
αCD25-APC	3C7	1:400	Biolegend	101910
αCD44-BV510	IM7	1:200	Biolegend	103044
αCD44-BV605	IM7	1:300	Biolegend	103047
αCD8a-BV421	53-6.7	1:200	Biolegend	100738
αCD8a-PE/Cy7	53-6.7	1:300	Biolegend	100722
αTCRß-FITC	H57-597	1:300	eBioscience	11-5961-85
αCD4-PE	RM4-4	1:300	Biolegend	116006
αCD4-A700	RM4-4	1:300	Biolegend	116022
αCD62L-PerCP/Cy5.5	MEL-14	1:300	Biolegend	104431
αCD127-BV421	A7R34	1:300	Biolegend	135023
αKLRG1(MAFA)-APC	2F1/KLRG1	1:100	Biolegend	138411
αCD11b-biotin	M1/70	1:250	Biolegend	101204
αGr1-biotin	RB6-8C5	1:250	Biolegend	108404
αTer119-biotin	TER119	1:250	Biolegend	116204
αCD45R/B220-biotin	RA3-6B2	1:250	Biolegend	103204
αNK1.1-biotin	PK136	1:250	Biolegend	108704

Table S3: Antibodies and stimulating components for CD4$^+$ T-cell isolation and T helper differentiation

**Biotinylated Antibodies for CD4$^+$ T-cell isolation**				
Antibody	Clone	Dilution	Supplier	Identifier
αCD11b	M1/70	1:200	Biolegend	101204
αTer119	Ter119	1:200	Biolegend	116204
αNK1.1	PK136	1:200	Biolegend	108704
αGr.1	RB6-8C5	1:200	Biolegend	108404
αCD8	53-6.7	1:100	Biolegend	100704
αB220	RA3-6B2	1:100	Biolegend	103204
αCD19	6D5	1:100	Biolegend	115504
**Reagents for T helper differentiation**				
Reagent	Clone		Supplier	Identifier
αCD3	145-2C11		BioXcell	BE0001-1
αCD28	37.51		BioXcell	BE0015-1
αIL-4	11B11		Invitrogen	14-7041-85
mouse IL-12			Peprotech	210-12
mouse IL-4			Peprotech	214-14
αIL-12	C18.2		Invitrogen	14-7122-81
αIFNγ	XMG1.2		Invitrogen	14-7311-85
IL-23			Peprotech	210-23
human TGFß			Gibco	PHG920
mouse IL-6			Peprotech	216-16
